# Proteomics of Protein Secretion by *Aggregatibacter actinomycetemcomitans*

**DOI:** 10.1371/journal.pone.0041662

**Published:** 2012-07-25

**Authors:** Vincent Zijnge, Thomas Kieselbach, Jan Oscarsson

**Affiliations:** 1 Oral Microbiology, Department of Odontology, Umeå University, Umeå, Sweden; 2 Department of Chemistry, Umeå University, Umeå, Sweden; University of Malaya, Malaysia

## Abstract

The extracellular proteome (secretome) of periodontitis-associated bacteria may constitute a major link between periodontitis and systemic diseases. To obtain an overview of the virulence potential of *Aggregatibacter actinomycetemcomitans*, an oral and systemic human pathogen implicated in aggressive periodontitis, we used a combined LC-MS/MS and bioinformatics approach to characterize the secretome and protein secretion pathways of the rough-colony serotype a strain D7S. LC-MS/MS revealed 179 proteins secreted during biofilm growth. Further to confirming the release of established virulence factors (e.g. cytolethal distending toxin [CDT], and leukotoxin [LtxA]), we identified additional putative virulence determinants in the secretome. These included DegQ, fHbp, LppC, Macrophage infectivity protein (MIP), NlpB, Pcp, PotD, TolB, and TolC. This finding indicates that the number of extracellular virulence-related proteins is much larger than previously demonstrated, which was also supported by *in silico* analysis of the strain D7S genome. Moreover, our LC-MS/MS and *in silico* data revealed that at least Type I, II, and V secretion are actively used to excrete proteins directly into the extracellular space, or via two-step pathways involving the Sec/Tat systems for transport across the inner membrane, and outer membrane factors, secretins and auto-transporters, respectively for delivery across the outer membrane. Taken together, our results provide a molecular basis for further elucidating the role of *A. actinomycetemcomitans* in periodontal and systemic diseases.

## Introduction

The destruction of the periodontium in periodontitis is the most common cause of tooth-loss worldwide. Periodontitis is a bacterially induced chronic inflammatory disease of the periodontium, which is also epidemiologically associated with systemic conditions such as cardiovascular diseases and rheumatoid arthritis. Periodontitis is associated with a defined subgingival microbial composition of the dental plaque biofilm, however the contribution of these bacteria to disease progression is poorly understood [1]–[4]. Hence, research in this area can improve the understanding of periodontitis and additional chronic diseases, and possibly lead to the identification of novel background mechanisms for increased cardiovascular risk. A commonly used model organism in periodontitis is the Gram-negative oral and systemic human pathogen *Aggregatibacter actinomycetemcomitans*, which is associated with aggressive periodontitis and endocarditis [5]–[7]. The virulence mechanisms executed by *A. actinomycetemcomitans* and their possible implication in periodontal and systemic disease are not clearly understood. The extracellular proteome, or secretome, of this organism is presumed to represent a key element. *A. actinomycetemcomitans* releases several factors that may play a role in modulating the host response, including leukotoxin (LtxA) [8], cytolethal distending toxin (CDT) [9], GroEL [10], peptidoglycan-associated lipoprotein (PAL) [11], and lipopolysaccharide (LPS) [12]. However, as a whole the secretome of this species is largely unexplored. Moreover, there is lacking knowledge regarding the set of secretion machineries that is used to deliver proteins to the exterior by *A. actinomycetemcomitans*. We and others have demonstrated that *A. actinomycetemcomitans* can deliver some of its virulence factors into human cells via outer membrane vesicles (OMVs) [13], [14]. Moreover, the species can excrete free-soluble surface components with proinflammatory activity independently of OMVs [15]. As both OMVs and free-soluble surface material are abundantly produced locally in the plaque biofilm, their potential entry into the circulation may constitute a significant source of inflammatory stimulants along with the planktonic bacteria in the bloodstream [16], [17]. To obtain a comprehensive overview of the potentially virulence-related extracellular proteins of *A. actinomycetemcomitans*, we have used liquid chromatography-tandem mass spectrometry (LC-MS/MS) to characterize the secretome of strain D7S (serotype a) during biofilm growth. Together with an *in silico* approach to identify the protein secretion machineries and candidate substrates for extracellular release in the strain D7S genome, this study provides a comprehensive overview of the active secretion pathways and a molecular basis for the pathogenic potential of *A. actinomycetemcomitans*.

## Results and Discussion

### Identification of Virulence-related Proteins in the *A. actinomycetemcomitans* Strain D7S Secretome

**Figure 1:**
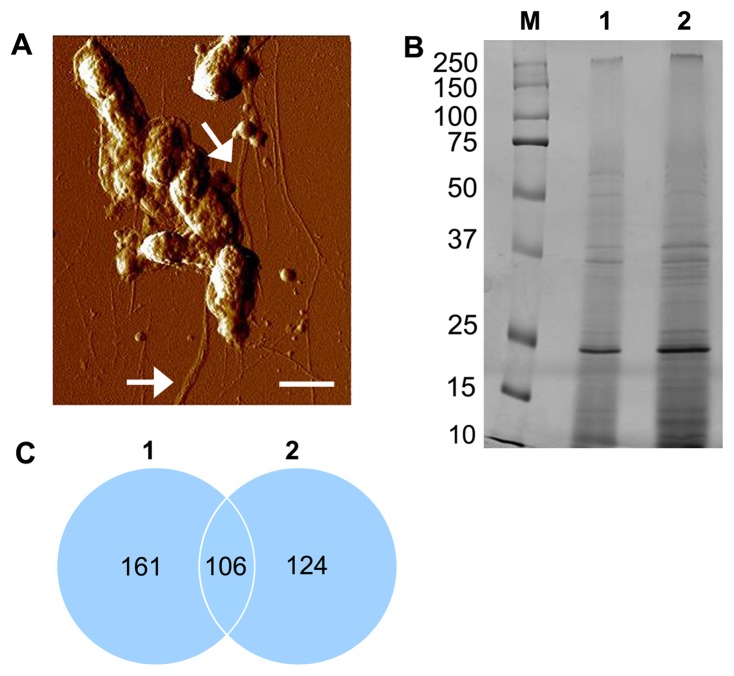
Analysis of the secretome of *A. actinomycetemcomitans* strain D7S during biofilm growth. (A) Atomic force micrograph of strain D7S grown as biofilm. Bundles of Flp pili are indicated by arrows. Bar = 700 nm. (B) Coomassie blue-stained SDS-PAGE of the two independent secretome preparations, denoted 1 and 2 (protein concentration ∼0.75 µg/µl; ∼20 µg protein applied on the gel), obtained from strain D7S biofilms, and which were analyzed in this study. The sizes (kDa) of the proteins in the prestained molecular mass marker (M) are indicated along the left side. (C) Venn diagram obtained from the comparison of the LC-MS/MS-identified proteins of the two strain D7S secretome preparations (1 and 2).

The rough-colony serotype a *A. actinomycetemcomitans* strain D7S was selected for this study as virulence properties of this strain and its derivatives have been frequently assessed in functional studies [14], [15], [18], [19], [20]–[22], and as its genome was recently characterized [23]. To identify the proteins extracellularly secreted by strain D7S, it was cultivated in biofilm form (Fig. 1A), and harvested at approximately early stationary phase (data not shown). Secretome preparations (∼20 µg protein; protein concentration ∼0.75 µg/µl) from two independent experiments were then analyzed by LC-MS/MS (Fig. 1B, and Fig. S1). Database searches (NCBInr) resulted in the identification of 179 proteins, out of which 106 were present in both of the secretome preparations (Fig 1C, and Table S1). This result is similar to an earlier study revealing approximately 120 proteins in the extracellular proteome of *A. actinomycetemcomitans* strain NCTC9710 (serotype c), albeit only five of the secreted proteins were identified [24]. The programs SosuiGramN, Cello 2.5 and PsortB were used to predict the subcellular localizations of the 179 proteins. According to our findings, 92 proteins were predicted to be cytoplasmic (51.4%), 40 periplasmic (22.3%), 19 located in the outer membrane (10.6%), and three to be extracellular (1.7%), whereas the subcellular locations of 25 (14%) proteins could not be predicted (Fig. S2, and Table S1). The relatively high abundance of proteins found by LC-MS/MS predicted to be either periplasmic or located in the outer membrane is consistent with the release of OMVs and free-soluble surface material by *A. actinomycetemcomitans* [15], [25]. OMVs are a common source of periplasmic and outer membrane proteins [26], and several are associated with, and secreted via *A. actinomycetemcomitans* OMVs [25]. The OMV’s (diameter 50–200 nm according to electron microscopy [15], [18]) are not expected to be filtered out (pore size 0.22 µm) during the preparation of extracellular protein extracts from strain D7S. Of the proteins identified by LC-MS/MS, 102 (57%) did not contain a signal sequence (Table S1). This is in the same range as similar studies with *Campylobacter concisus* and *Listeria* spp., respectively, which revealed that 57% [27] or 49% [28] of the extracellular proteins lacked a signal sequence. Identification of cytoplasmic proteins in the extracellular fraction may be a result of cell lysis or cell autolysis occurring during growth [29]. However, among the proteins identified by LC-MS/MS (Table S1) we did not find cyclic AMP receptor protein (CRP; GI:293390977). This cytoplasmic protein was used as a lysis marker in our earlier studies assessing the release of proteins by strain D7S grown as biofilm [15], [18]. Notably, CRP was not detected in extracellular supernatants of D7S cultivated as biofilm for up to 3 days, but could be released upon deliberate lysis of the *A. actinomycetemcomitans* cells [15], [18]. Hence, the absence of CRP in our present secretome preparations would argue against release due to bacterial lysis. An alternative explanation why proteins without signal seqeuence appeared in the secretome preparations may be non-classical secretion, *e.g.* translocation via hitherto uncharacterized routes for protein transport [30]. There is substantial evidence of cytoplasmic proteins being incorporated into OMVs of different bacterial species [31], [32]. Moreover, it has been suggested that certain cytoplasmic proteins may in fact have dual functions and can be targeted by the cell to different subcellular sites [33].

To assess the virulence potential of the strain D7S secretome, all proteins identified by LC-MS/MS (n = 179) were manually searched for their earlier reported associations with virulence-related activity in *A. actinomycetemcomitans* or, when applicable, in other organisms. From this screening, 26 proteins were of particular interest (Table 1). In accordance with previous studies, the secretome included several (n = 17) proteins demonstrated earlier to contribute to the pathogenicity of *A. actinomycetemcomitans*: CdtA (GI:293392175), CdtB (GI:293392176), and CdtC (GI:293392177) constitute a tripartite (CdtABC) genotoxin, produced by several Gram-negative organisms, and which can induce G_2_ cell cycle arrest, progressive cellular distention, and/or apoptosis in many cell types [34], [35]. The CdtB protein acts as a type I DNase [36], [37], whereas CdtA and CdtC are involved in host cell recognition and internalization of CdtB in host cells [38], [39]. The chaperonin GroEL (GI:293391167) can activate a plethora of mammalian cells, including macrophages, keratinocytes and periodontal ligament epithelial cells [10], [15], [40]. LtxA (GI:293390491) is a well-studied virulence determinant in *A. actinomycetemcomitans* that contributes to pathogenesis by killing lymphoid and myeloid cells [41]–[44]. Macrophage infectivity protein (MIP; GI:293391100) is a surface-exposed lipoprotein that is involved in intracellular survival and persistence of several species, including *A. actinomycetemcomitans*, *Legionella pneumophila* and *Neisseria* spp. [45]–[48], and which meets several important criteria for a potential meningococcal vaccine antigen [49]. The outer membrane proteins (OMP) Omp18/16 (GI:293391272), Omp29 (GI:293390286; also referred to as OmpA and Omp34), Omp39 (GI:293391956), Omp100 (GI:293390333; also referred to as ApiA), and YaeT (GI:293390810) exhibit antigenicity in the host [50]–[53]. Notably, Omp39 and YaeT are antigenic in patients carrying the highly leukotoxic *A. actinomycetemcomitans* JP2 clone [52], and Omp29 is associated with the entry of *A. actinomycetemcomitans* into gingival epithelial cells [50]. Omp64 has been suggested to play a role in iron acquisition [51]. It cannot be excluded that the Omp29-(OmpA)-like protein (GI:293390327; ∼76% amino acid identity to Omp29) could also exhibit antigenicity in the host similar to *A. actinomycetemcomitans* Omp18/16, Omp29, Omp39, and Omp100. PAL (GI:293390507) is a lipoprotein that has proinflammatory activity *in vitro* [11], [15], [18]. RcpA (GI:293391332), TadD (GI:293391326), TadE (no assigned GI in the D7S genome database used; see also Type II secretion below), and TadG (GI:293391324) are part of the *A. actinomycetemcomitans tad* (tight adherence) gene locus (*flp-1*, *flp-2*, *tadV*, *rcpCAB*, *tadZABCDEFG*) [54] encoding a macromolecular machinery for Flp pili biogenesis [55], [56], which is required for virulence in a rat model for periodontal disease [57]. Whereas studies on *Porphyromonas gingivalis* fimbriae have indicated their modulation of signaling pathways mediating proinflammatory or proadhesive effects [58], such function of *A. actinomycetemcomitans* fimbriae or fimbrial components has not yet been experimentally demonstrated. To corroborate our LC-MS/MS results, we used immunoblotting with specific antibodies to confirm the presence of selected of the above proteins in the extracellular medium of strain D7S biofilm samples used for the LC-MS/MS analysis (Fig. S3). Extracellular release of GroEL and PAL by strain D7S grown as a biofilm is consistent with our published data [15].

**Table 1:** Virulence-related proteins in the *A. actinomycetemcomitans* strain D7S secretome identified by LC-MS/MS.

GI accessionnumber D7S	Protein namea)	Signalsequenceb)	COG functionalclassificationc)	Secretomepreparationd) 1	Secretomepreparationd) 2
**Earlier reported association with virulence-related activity in ** ***A. actinomycetemcomitans*** e)					
293391167	Chaperonin GroL	n	Post-translational modification, proteinturnover, chaperone functions	y	y
293392175	Cytolethal distending toxin protein A	y	uncategorized	n	y
293392176	Cytolethal distending toxin protein B	y	uncategorized	y	y
293392177	Cytolethal distending toxin C	y	uncategorized	y	n
293391326	Flp pilus assembly protein TadD	y	Intracellular trafficking and secretion	y	y
32452630f)	Flp pilus assembly protein TadE	n	uncategorized	y	n
293391324	Flp pilus assembly protein TadG	n	Intracellular trafficking and secretion	y	y
293390491	Hemolysin Ag)	n	uncategorized	y	y
293391100	Macrophage infectivity protein	y	Post-translational modification, proteinturnover, chaperone functions	y	y
293390286	OmpA-like outer membrane proteinh)	y	Cell wall/membrane/envelope biogenesis	y	y
293391272	Outer membrane protein 18/16	y	uncategorized	y	y
293391956	Outer membrane protein 39	y	Cell wall/membrane/envelope biogenesis	y	y
293390533	Outer membrane protein 64	y	Inorganic ion transport and metabolism	y	n
293390333	Outer membrane protein 100i)	y	uncategorized	y	y
293391100	Outer membrane protein assemblycomplex, YaeT protein	y	Cell wall/membrane/envelope biogenesis	y	y
293390507	Peptidoglycan-associated lipoprotein	n	Cell wall/membrane/envelope biogenesis	y	n
293391332	Type II/IV secretion system secretinRcpA/CpaC	y	Intracellular trafficking and secretion	y	y
**Earlier reported association with virulence-related activity in other organisms but not yet in ** ***A. actinomycetemcomitans*** e)					
293390346	Lipoprotein LppC protein	y	General Functional Prediction only	y	y
293391894	NlpB protein	y	Cell wall/membrane/envelope biogenesis	y	y
293390327	Outer membrane protein Aj)	y	Cell wall/membrane/envelope biogenesis	y	y
293392122	Outer membrane lipoprotein Pcp	y	Cell wall/membrane/envelope biogenesis	y	n
293390241	PotD protein	y	Amino Acid metabolism and transport	y	n
293390325	Protease DegQ	y	Post-translational modification, proteinturnover, chaperone functions	y	y
293391790	Putative lipoproteink)	y	uncategorized	y	y
293390508	Tol-Pal system propeller repeat proteinTolB	y	Inorganic ion transport and metabolism	y	y
293389951	TolC protein	y	Intracellular trafficking and secretion	y	y

a) Full name of D7S genome database entry.

b) Presence (y) or absence (n) of N-terminal signal sequence.

c) Functional classification according to the database of Clusters of Orthologous Groups of proteins (COGs).

d) Indicates whether the protein was identified by LC-MS/MS (y) or not (n) in each of the two secretome preparations.

e) See text.

f) Not found in the D7S genome database used.

g) Also referred to as leukotoxin or LtxA.

h) Also referred to as Omp29 and Omp34.

i) Also referred to as ApiA.

j) This protein exhibits 76% amino acid identity with Omp29.

k) This protein exhibits >90% amino acid identity with fHbp of *Neisseria* spp.

Further to the above proteins, the D7S secretome included eight additional proteins that could potentially contribute to the pathogenicity of *A. actinomycetemcomitans* (Table 1): DegQ (GI: 293390325) is a periplasmic protease, which has been suggested to play a protective role when *E. coli* enters a host organism [59]. In *Actinobacillus pleuropneumoniae*, LppC (GI:293390346) is an outer membrane lipoprotein that can induce a specific antibody response in pigs [60]. NlpB (GI:293391894; also referred to as DapX) is an outer membrane lipoprotein that was demonstrated to be essential for virulence of *Yersinia pestis* in murine models of bubonic and septicemic plague [61]. Pcp (GI:293392122) is a lipoprotein highly abundant on the surface of *Y. pestis*, and has been hypothesized to be important for pathogenicity [62]. PotD (GI:293390241) is a surface-associated spermidine- and putrescine-binding protein in *Streptococcus pneumoniae* and a virulence factor in murine models of systemic and pulmonary infection [63]. TolB (GI: 293390508) was identified in a genome-wide screen of *Salmonella typhimurium* as a virulence factor in mice contributing to resistance to deoxycholate and serum and bacterial survival in J774 cells [64]. TolC (GI: 293389951) is involved in multidrug resistance and plays a key role in virulence in several Gram-negative organisms [65]–[68]. Finally, a putative 28-kDa lipoprotein **(**GI:293391790**)** shows strong similarity (>90%) with the factor H-binding protein (fHbp) of *Neisseria* spp. and contains the conserved lipoprotein C domain (pfam08794). Factor H-binding protein attaches to the human regulatory protein factor H that regulates homeostasis of the complement system. By binding factor H, bacteria mimic host tissue and avoid complement initiated cell lysis [69]. The novel observation that *A. actinomycetemcomitans* possess an fHbp homologue may explain the finding that complement-mediated phagocytosis of *A. actinomycetemcomitans* is generally inefficient [70].

### Identification of Virulence-related Proteins among the Proteins Predicted to be Secreted by *A. actinomycetemcomitans* Strain D7S

In the present study we assessed the secretome composition during biofilm growth using LC-MS/MS. It cannot be excluded that some proteins targeted for secretion may have been missed due to the experimental setup and analysis methods used. For instance, previous analysis of 2D-PAGE protein patterns during various growth conditions revealed differential protein levels for up to 8% of the secreted subproteome of *A. actinomycetemcomitans* strain NCTC9710 [24]. To identify such proteins and to gain further insight into the pathogenic potential of this organism, we therefore determined the theoretical secretome by *in silico* analysis of the strain D7S genome using a rational approach formalized in a bioinformatics workflow [71]. In this workflow, proteins with a signal peptide and proteins predicted to be extracellularly secreted using any of the software packages SosuiGramN, Cello 2.5 and PsortB were identified. From the identified proteins, those with at least two predicted alpha helical transmembrane domains were regarded as putatively attached to the inner membrane and discarded from the list of proteins. Hence, the final list contained 250 proteins having the potential to be extracellularly secreted. Out of these, 73 proteins (29.2%) were detected by LC-MS/MS. The 250 proteins were grouped into lipoproteins and outer membrane proteins forming beta barrels, respectively (Table S2A, and Table S2B). The remaining proteins were categorized according to their predicted subcellular localization (Table S2C). Analogously to the proteins identified by LC-MS/MS, the 250 proteins found by *in silico* analysis were manually screened for their earlier reported associations with virulence-related activity in *A. actinomycetemcomitans* or, when applicable, in other organisms. As summarized below, this screening revealed seven proteins not identified by LC-MS/MS, which were of particular interest (Table 2).

**Table 2:** Additional virulence-related proteins identified among the proteins predicted to be secreted by *A. actinomycetemcomitans* strain D7S.

GI accession number D7S	Protein namea)	Signal sequenceb)	COG functional classificationc)
**Earlier reported association with virulence-related activity in ** ***A. actinomycetemcomitans*** d)			
293391897	Autotransporter adhesin Aae	y	uncategorized
293391439	Chb proteine)	y	Carbohydrate metabolism and transport
293390626 293390627 293390628f)	Extracellular matrix protein adhesin A	n	Intracellular trafficking and secretion
**Earlier reported association with virulence-related activity in other organisms but not yet in ** ***A. actinomycetemcomitans*** d)			
293391047	Lipoprotein VacJ	y	Cell wall/membrane/envelope biogenesis
293391875	Opacity-associated protein OapA	n	Chromatin Structure and dynamics
293391876	Opacity-associated protein OapB	y	uncategorized
293391443	Outer membrane antigenic lipoprotein B	y	Cell envelope biogenesis, outer membrane

a) Full name of D7S genome database entry.

b) Presence (y) or absence (n) of N-terminal signal sequence.

c) Functional classification according to the database of Clusters of Orthologous Groups of proteins (COGs).

d) See text.

e) Also referred to as dispersin B or DspB.

f) Assigned as three separate proteins in the D7S genome database used.

#### Lipoproteins

Proteins containing a lipobox are translocated across the inner membrane in a Sec dependent way and can remain attached to the inner membrane or guided to the outer membrane by the LolCDE complex [72] where they can become exposed to the exterior or secreted. *In silico* analysis of the D7S genome revealed 71 proteins containing a lipobox, out of which 23 were present in the secretome as determined by LC-MS/MS (Table S2A). Among the proteins not found by LC-MS/MS, four could have a potential to contribute to *A. actinomycetemcomitans* virulence. DspB (Dispersin B; also referred to as Chb [GI:293391439]) is a glycoside dehydrogenase that degrades poly-N-acetylglucosamine, the main component of the *A. actinomycetemcomitans* biofilm matrix. By degrading the matrix, DspB is involved in dispersion of *A. actinomycetemcomitans* cells from the attached biofilm [73]. OapB (GI:293391876) belongs to a growing family of lysozyme inhibitors contributing to lysozyme tolerance in Gram-negative bacteria [74]. This function may be beneficial for survival in the oral cavity where lysozyme is an important component of the antibacterial activity of saliva. The outer membrane antigenic lipoprotein B (GI:293391443) exhibits ∼80% amino acid sequence similarity to the lipoprotein NlpD of *Yersinia pestis*, which is essential for the development of bubonic and pneumonic plague in mice [75]. Finally, VacJ (GI:293391047) is exposed on the surface of *Shigella flexneri* cells, and is essential for the spreading of this species through the intercellular spaces of tissues and, moreover, induces protrusions of eukaryotic cells upon expression in intracellular bacteria. This facilitates migration of the bacteria to the cytoplasm of the next cell [76].

#### Beta barrel forming proteins

Using the BCCS and TMB-Hunt packages with the BCCS >3 and/or TMB-Hunt ≥4 criteria, 35 beta barrel forming proteins were found to be encoded by the D7S genome, including 11 that were identified by LC-MS/MS (Table S2B). Among the proteins not identified by LC-MS/MS, EmaA (GI293390626, GI293390627, and GI293390628) is a candidate virulence-related protein that could promote adhesion of *A. actinomycetemcomitans* [77].

#### Predicted subcellular localization of the remaining proteins based on a most-votes analysis

The subcellular localization of the remaining 144 proteins identified by *in silico* analysis, containing 39 identified by LC-MS/MS, could not be predicted solely based on the presence of a signal peptide or secondary structure. Therefore a majority of votes analysis was used to categorize them (Table S2C). We are aware of that the subcellular localization prediction tools should be used with caution. This is exemplified by the 53 proteins for which no subcellular localization could be predicted, and for the ten cytoplasmically predicted proteins that nevertheless do contain a signal peptide. Among the proteins not detected by LC-MS/MS, Aae (GI:293391897) modulates binding of *A. actinomycetemcomitans* to human buccal epithelial cells [78], and OapA (GI:293391875) is a surface-exposed protein in *Haemophilus influenzae* that mediates epithelial adhesion via an unknown receptor [79].

### Delineation of Active Protein Secretion Pathways in *A. actinomycetemcomitans* Strain D7S

**Figure 2:**
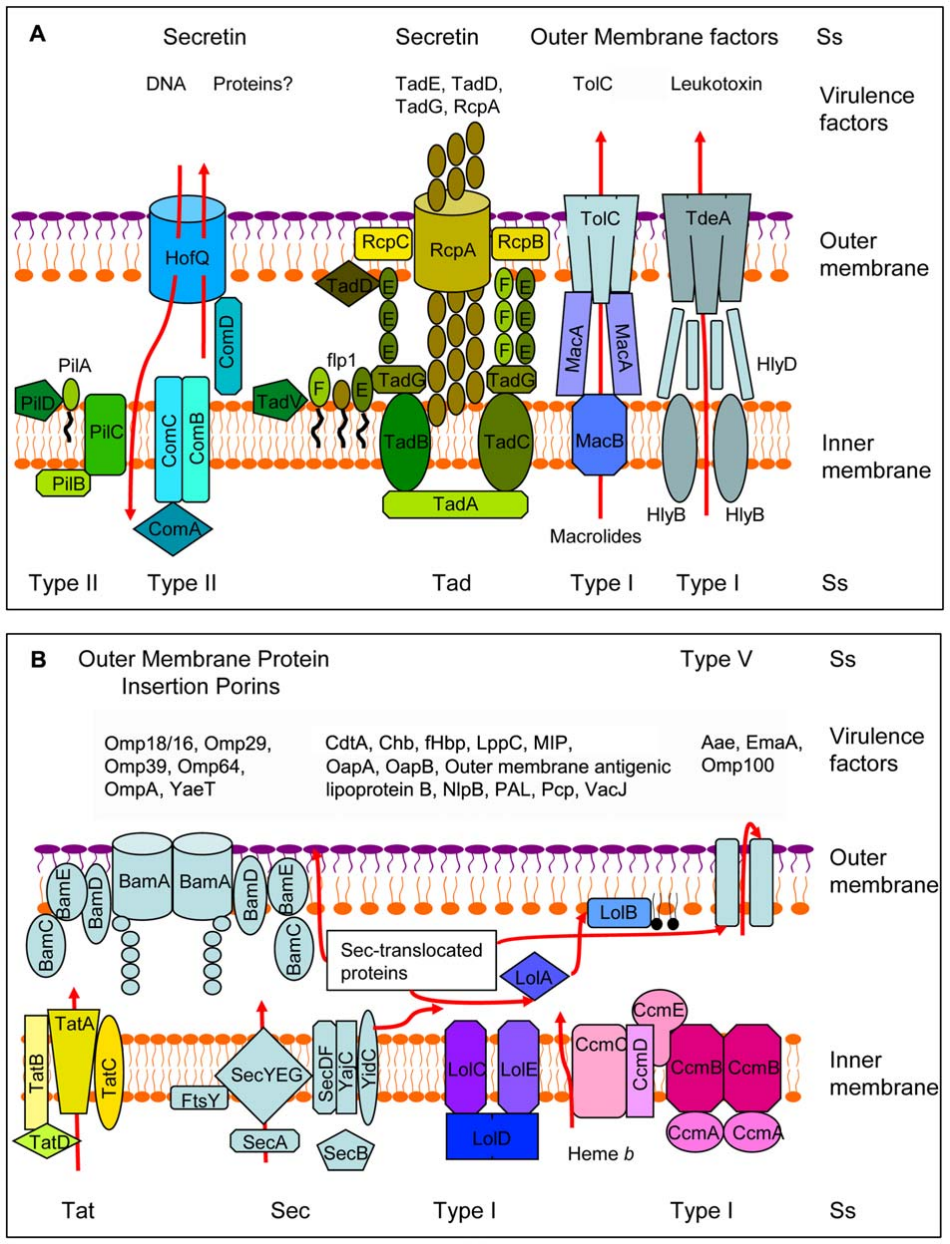
Schematic representation of functionally active protein secretion systems (Ss) present in *A. actinomycetemcomitans* strain D7S. (A) Secretin and OMF-related secretion systems. (B) OmpIP and Type V-related secretion systems. Secretion systems are matched with a selection of their substrates among the virulence-related proteins identified by LC-MS/MS analysis and/or by *in silico* analysis of the strain D7S genome.

After having assessed the virulence potential of the *A. actinomycetemcomitans* strain D7S secretome by two complementary approaches, we next wanted to disclose the active pathways for protein secretion of this strain. Blast searches for secretion systems as defined in the Transporter Classification Database (TCDB) revealed the presence of homologues to Sec, Tat, Type I, Type II, and Type V secretion systems in the D7S genome. Moreover, we identified secretins, the Outer Membrane Factor, and Outer Membrane Protein Insertion Porins (TCDB classification numbers indicated in corresponding sections below). On the other hand, Blast searches against typical Type III (#3.A.6), Type IV (#3.A.7), Type VI (#9.A.34) or Type VII (#1.C.95, #9.A.44, #9.A.25) proteins revealed no homologues to these secretion systems in the D7S genome. The functionality of the identified protein secretion systems encoded by strain D7S was then primarily assessed by matching them with their substrates among the 179 secretome proteins identified by LC-MS/MS. Our findings regarding the respective secretion systems are summarized below, in Fig. 2, and in Table S3. As indicated in Table S3, in some cases a protein was found to have several gene identifiers in the D7S genome database used.

#### General secretory pathway (# 3.A.5)

Genetic determinants encoding general secretory pathway (Sec) components, a major pathway for translocation of proteins across the inner membrane, was found in the strain D7S genome (Table S3A). Proteins designated for secretion by the Sec pathway contain a specific N-terminal signal sequence that directs them to the appropriate machinery [80]. Among the secretome proteins, 74 (41.3%) were found to carry such N-terminal signal sequence (Table S1), indicating that they are targeted for secretion via the general secretory pathway. This is consistent with a functionally active general secretory pathway in *A. actinomycetemcomitans* strain D7S.

#### Twin arginine pathway (# 2.A.64)

Homologues to TatABC were identified in the *A. actinomycetemcomitans* D7S genome (Table S3A). The Tat translocase is composed of two or three inner membrane located proteins, TatA, TatB and TatC. The Tat secretion machinery is dedicated to the translocation of folded proteins across the membrane, which is a clear distinction from the Sec translocation pathway that translocates unfolded peptides [80]. Substrates targeted for the Tat pathway have an N-terminal signal sequence that resembles the NHC signal sequence organization also found in Sec-directed substrates. The distinguishing feature of Tat-directing signal sequences is the consensus motif (ST)-R-R-X-F-L-K in the hydrophobic domain. Among the extracellularly secreted proteins identified by LC-MS/MS, three appeared to contain a Tat signal sequence (Table S1). This suggests that the Tat pathway was functionally active during the present experimental setup.

#### Type I (ATP binding cassette, # 3.A.1)

Our database searches revealed 53 proteins encoded by *A. actinomycetemcomitans* strain D7S having an ABC transporter domain (PS50893). Of these proteins, 16 are involved in secretion whereas the rest function as substrate uptake systems (Table S3B). *In silico* analysis revealed five systems for biosynthesis purposes, *e.g.* the transport of LPS (Wzm/Wzt and LptABCDEFG), the periplasmic cytochrome biosynthesis (CydCD and CcmABCDE) and lipoprotein (LolABCDE) transport to the outer membrane. Other proteins are part of a Type I secretion system that is dedicated to secrete a substrate to the exterior. These systems are LtxCABD, MacAB, a putative exporter involved in toluene tolerance, MdlB (GI:293390739), and exporters with unknown function (GI:293389953, 293390276, 293391310). LtxA is the secreted substrate of the LtxCABD system. Our LC-MS/MS data revealed the extracellular release of LtxA and several additional ABC transporters during the growth conditions applied (Table S1), which is consistent with a functionally active Type I secretion in strain D7S.

#### Type II (Main terminal branch; # 3.A.15)

Our *in silico* analysis of the *A. actinomycetemcomitans* strain D7S genome also revealed the presence of several Type II secretion-related genes (Table S3C). Type II secretion systems are protein complexes that mediate the translocation of Sec- or Tat-secreted proteins across the outer membrane, and share homology with Type IV pilus production (T4P) and competence systems [81], [82], [83]. In *A. actinomycetemcomitans*, a Type IV pilus (T4P)-like gene cluster named PilABCD has been identified that is involved in natural competence but not in pilus formation [21]. However, that study did not reveal the identity of the outer membrane protein that forms the pore for DNA uptake. An upstream gene cluster that is also involved in competence, ComABCD [84], encodes the HofQ secretin. Hence, it cannot be excluded that HofQ may function as a secretin needed for DNA uptake via PilABCD. The close resemblance of the proteins involved in T4P, T2SS and competence has led others to hypothesize a system in which DNA binding and uptake is mediated by a pilus (or pseudo-pilus) structure that combines T4P and competence-specific pili [85]. Whether PilABCD is part of a T4P or a T2SS and thereby involved in secretion is unknown but it might be a potential candidate secretion system to deliver Sec translocated proteins across the outer-membrane.

#### Type II (Tight adherence [Tad]; # 3.A.7.15.1)

The *tad* gene cluster in *A. actinomycetemcomitans* (*flp-1*, *flp-2*, *tadV*, *rcpCAB*, *tadZABCDEFG*) encodes long filamentous fimbrils composed of bundled Flp pili [54], [86], and has been classified as a novel lineage of Type II secretion [56]. The function of this macromolecular transport system in *A. actinomycetemcomitans* and other species is not entirely understood. It has been suggested that the pseudopilins TadE and TadF form an oligomeric structure in the periplasm, which could guide pilus assembly and provide contact with the outer membrane RcpA secretin complex [56]. Expression of the Tad-pilus is important for the characteristic rough-colony morphology of *A. actinomycetemcomitans* strains including D7S [20], [87]. Strain D7S encodes a complete *tad* gene locus, and mutational analysis has revealed that fimbriae expression requires the function of *flp-1*, *rcpA*, *rcpB*, *tadB*, *tadD*, *tadE*, and *tadF* [22]. The concerted finding of several Tad locus proteins (TadD, TadE, TadG and RcpA) in the strain D7S secretome (Table S1), and the production of bundle forming pili by D7S [20](Fig. 1A) is consistent with functionally active Type II secretion. However, notably our BLAST searches of the strain D7S genome database failed to detect either *flp-2* or *tadE* (Table S3C) despite that these genes were earlier characterized in this strain [22]. The reason for this discrepancy is not known.

#### Type IV (# 3.A.7)

Eleven *A. actinomycetemcomitans* Type IV secretion system (T4SS) determinants were earlier found to be encoded on a plasmid (pVT745) in one strain (VT745), whereas they were present on the chromosome of a different strain (VT747) [88], [89]. Homologues to these T4SS determinants are encoded on the conjugative plasmid S57 (92% nucleotide identities to pVT745) [90]. One additional gene (GI: 1243302) is located within the gene cluster and may serve as an entry exclusion protein that is thought to inhibit DNA transfer after mating pairs have been formed [88], [91]. Also the novel plasmid S25 harbours a set of ten T4SS related proteins [90]. One gene (GI: 8537963) is probably a VirB3/B4 fused protein. However neither of the plasmids pVT745, S25 or S57 is carried by strain D7S [23]. Moreover, our *in silico* analysis revealed no T4SS related genes or putative T4SS protein substrates such as CagA, CagE, and VirB1 [92], [93] to be encoded on the D7S chromosome. This is consistent with our LC-MS/MS data.

#### Type V (# 1.B.12, # 1.B.40, # 1.B.54)

A Prosite search with PS51208 for the autotransporter beta domain revealed only Aae as a possible autotransporter in the *A. actinomycetemcomitans* D7S genome (Table S3C). Literature searches showed that EmaA [77] and Omp100 [78] are also considered to be autotransporters in *A. actinomycetemcomitans*. This assumption was based on the presence of a C-terminal YadA region and the predicted formation of beta barrels. EmaA and Omp100 have thereby been assigned as autotransporters of the AT-2 family, but lack the typical autotransporter beta domain. Due to the absence of an autoproteolytic domain, Aae, Omp100 and EmaA are most likely exposed on the cell surface. Indeed, Aae and EmaA have been shown to form surface structures on *A. actinomycetemcomitans* cells and are involved in adhesion to buccal epithelial cells and collagen [77], [94]. Identifying Omp100 among the extracellular proteins (Table S1) is consistent with functionally active Type V secretion in strain D7S.

#### Secretins (# 1.B.22)

In our *in silico* analysis of the *A. actinomycetemcomitans* strain D7S genome, we identified HofQ and RcpA as proteins that carry one and two of the secretin consensus sequences, respectively [95] (Table S3C). RcpA is essential for Tad-pilus formation and forms the pore through which the pilus extends through the outer membrane [56], [96]. Production of bundle forming pili by strain D7S [20](Fig. 1A) is consistent with the RcpA secretin being functionally active. HofQ shares homology with the competence protein ComE of *Haemophilus influenzae* and the *Pseudomonas aeruginosa* PilQ protein involved in type II secretion/type IV pilus biogenesis. HofQ may function in natural transformation, fibronectin binding and Type IV pilus biogenesis [97], although the exact mechanism is unclear.

#### Outer membrane factor (OMF) (# 1.B.17)

The TolC family of outer membrane proteins is ubiquitous among Gram-negative organisms and their role in protein secretion has been thoroughly characterized [98]. The archetypical TolC protein acts as an outer membrane factor by forming a pore, and is recruited by ABC transporters to export substrates to the exterior [99]. Hitherto, TdeA (GI: 293392240), also referred to as TolC [100], has been the only Outer Membrane Factor recognized in *A. actinomycetemcomitans*, and is a component of a drug efflux pump that plays a role in LtxA secretion [101]. Interestingly, our BLAST searches with OMF proteins against the strain D7S genome revealed the presence of one additional, tentative OMF, denoted TolC (GI: 293389951) (Table S3C), exhibiting ∼24% amino acid identity to TdeA. Albeit the putative role of this novel OMF in protein secretion remains to be experimentally confirmed, the recognition of TdeA, TolC, and several ABC transporters in the D7S secretome (Table S1) would be consistent with both OMF proteins playing an active role.

#### Outer membrane protein insertion porins (# 1.B.33)

The Outer Membrane Protein Insertional Porin (OmpIP) or Beta barrel Assembly Machinery (BAM) is responsible for the folding and insertion of outer membrane proteins in the outer membrane [102], and its functional activity in D7S is consistent with the recognition of several OMPs in the secretome (Table S1). In the strain D7S genome we found homologues to three BAM chaperones, and to BamA, BamC, BamD and BamE (Table S3C). However, similar to genomic analysis of *Neisseria* spp. [103], [104], we did not find a BamB homologue in the D7S genome, or in any other *A. actinomycetemcomitans* genomes available at the databases in NCBI. Studies in *E. coli* suggest that absence of BamB may hamper the correct insertion of outer membrane proteins and compromise the outer membrane permeability [105]. Hence, analogously to the hypothesis regarding *Neisseria* spp. [104], BamC may compensate for lack of BamB in *A. actinomycetemcomitans*. This remains to be tested.

### Concluding Remarks

In the present work we have assessed the virulence-potential of the extracellular proteome of the *A. actinomycetemcomitans* serotype a strain D7S using two complementary approaches, LC-MS/MS analysis of the secretome during biofilm growth, and *in silico* analysis of the D7S genome. Although the virulence potential of *A. actinomycetemcomitans* may vary among strains, these approaches together underscored that this organism releases a much larger arsenal of virulence-related proteins than previously demonstrated. Moreover, combining our LC-MS/MS and *in silico* data revealed active use of at least Type I, II, and V secretion to translocate proteins directly or via two-step pathways into the extracellular space. This includes the Sec/Tat systems for transport across the inner membrane, and outer membrane factors, secretins and auto-transporters for delivery across the outer membrane. The possible presence of non-classical protein secretion in *A. actinomycetemcomitans*, the formation of outer membrane vesicles and the unknown sorting pathway of lipoproteins to and across the outer membrane emphasizes the importance of combining *in silico* data with experimental evidence. We conclude that our present results provide a molecular basis for further disclosing the role of *A. actinomycetemcomitans* in periodontal and systemic disease. For instance, studies investigating the expression, secretion and function of novel putative extracellular virulence factors such as DegQ, fHbp, LppC, MIP, NlpB, Pcp, and PotD may provide new evidence how bacterial modulation of host cytokine expression repertoires may lead to destructive inflammation.

## Materials and Methods

### Bacterial Strains and Growth Conditions

The *A. actinomycetemcomitans* serotype a rough-colony strain D7S, and its *pal* mutant derivative, D7S-p [18] were used in this study. Strain D7S was originally isolated from a patient with aggressive periodontal disease [20]. The strain D7S was kindly donated by Dr. Casey Chen, University of Southern California. This strain has since been used in several of our earlier studies that were cited in our present work [14], [15], [18]. The strains were cultured on blood agar plates (5% defibrinated horse blood, 5 mg hemin/l, 10 mg Vitamin K/l, Columbia agar base) incubated in air supplemented with 5% CO_2_, at 37°C for 3 days as previously described [18]. For biofilm growth, 2×10^8^ bacterial cells were inoculated in 2 ml tryptic soy broth (Difco) in 24-well cell culture plates (Nunc), which were incubated in static culture in air supplemented with 5% CO_2_, at 37°C for 42 h. To assess the growth of *A. actinomycetemcomitans* biofilms, biofilm samples were stained with crystal violet and the amount of bound dye, which is proportional to the biofilm mass was quantitated by measuring its absorbance at 590 nm.

### Preparation of the *A. actinomycetemcomitans* Strain D7S Secretome

Following biofilm cultivation, 2 ml of the growth medium of a single well was carefully collected and then centrifuged for 10 min at 10.000×*g* to pellet down remaining bacterial cells. Supernatants were then filtered through 0.45 µm and subsequently 0.22 µm membranes prior to being desalted and concentrated into 120 µl H_2_O with Pall 10 K membrane filters according to the manufacturer’s instructions (Pall Corporation). Protein concentrations were determined using the Bradford Reagent (Sigma-Aldrich).

### SDS-PAGE and Western Immunoblotting

The quality of secretome preparations (protein concentration ∼0.75 µg/µl) was confirmed by running a fraction (∼5 µg protein) of the sample on an 8–16% linear gradient SDS-PAGE gel (Criterion, Bio-Rad). As control samples in immunoblots, *A. actinomycetemcomitans* whole cell preparations (∼1 µg/µl protein) equivalent to 10 µg protein were loaded on the gels. The procedures used for SDS-PAGE and immunoblot analysis have been described previously [11], [106]. Gels were stained using non-ammonical Silver-staining (BioRad). For immunoblot detection, we used polyclonal antisera raised in rabbits specific for *E. coli* GroEL (Sigma-Aldrich) and *A. actinomycetemcomitans* PAL [107]. The antisera were used at a final concentration of 1∶8000 and 1∶10.000, respectively. As secondary antibody, anti-rabbit horseradish peroxidase (HRP)-conjugate was used (1∶10.000). Immunoreactive bands were visualized using SuperSignal® (Pierce) and the ChemiDoc™ XRS + System (Bio-Rad).

### Atomic Force Microscopy

For atomic force microscopy, bacterial cells were suspended in ultrapure water (Millipore) and 10 µl bacterial suspension was then placed on a freshly cleaved mica surface. The samples were incubated for 5 min at room temperature and blotted dry before being placed into a desiccator for at least 2 h. Imaging was performed using a Nanoscope V Atomic Force Microscope (Bruker AXS) using Tapping Mode with standard silicon cantilevers. Final images were plane fitted in both x and y axes and are presented in amplitude mode.

### LC-MS/MS Analysis and Data Processing

For mass-spectrometry, protein samples equivalent to approximately 20 µg protein (protein concentration ∼0.75 µg/µl) were separated in 12 cm long 12% SDS-PAGE gels [106] containing 2 M urea. Subsequent to electrophoresis the gels were fixated using 10% acetic acid, 30% ethanol and stained using hot Coomassie blue [108]. In-gel digestion of peptides for analysis by mass spectrometry was carried out essentially as described earlier [109]. LC-MS/MS analysis of peptides was performed using an HCT-Ultra ETD II ion trap mass spectrometer (Bruker) linked to an Easy-nLC system (Proxeon). Spectra were acquired using the enhanced scanning mode covering a mass range from *m/z* 400 to *m/z* 1300. The LC separation of peptides was performed using a 5 µm C18 column (375 µm OD/75 µm ID×10 cm) (NanoSeparations) and a 60 min gradient ranging from 1 to 50 percent of acetonitrile. The flow rate was 300 nl min^−1^. The LC-MS/MS datasets were processed using Bruker DataAnalysis 4.0 SP4. Database searches using the peaklist files of the processed mass spectra were performed in the bacterial section of the NCBInr database using ProteinScape 2.1 (Bruker) and in-house licenses of Mascot 2.3.01 (www.matrixscience.com) and of Phenyx 2.6 (www.genbio.com). The search parameters allowed for one missed cleavage site and a mass error of 0.3 Da for both the MS and MS/MS mode. In addition, variable modifications including methionine oxidation, N-terminal acetylation, and derivation of cysteine by propionamide were considered. The Mascot.dat files of the database searches were submitted to the EBI-Pride repository (accession numbers: 22453–22456). Non-redundant protein lists were compiled from the database searches using the ProteinExtractor of ProteinScape 2.1 and settings for spectra acceptance as follows: Mascot score >100 and at least one peptide with a peptide ion score >55. Peptides with a Mascot ion score <30 were ignored. As for Phenyx scores, the minimum threshold for protein acceptance was 18 and at least one peptide with a score of 10 as required. Peptides with a Phenyx score <7 were not considered.

The non-redundant protein lists created by the ProteinExtractor were further inspected manually to ensure that each protein identification was based on at least two different peptide identifications. It is an intrinsic feature of the database searches than the identified proteins not only included hits from the sequenced genomes of *A. actinomycetemcomitans* strain D7S but also from strain D11S and from the partly sequenced genomes of additional *A. actinomycetemcomitans* strains present in the NCBI nr database. As for the protein identifications obtained from other strains than D7S, BLAST searches were performed to identify the corresponding D7S homologues.

### *In Silico* Analysis

The whole genome shotgun sequence of *A. actinomycetemcomitans* strain D7S with accession number ADCF00000000 [23] was downloaded from the European Bioinformatics Institute (http://www.ebi.ac.uk/), and used in this work. During the analyses a number of annotations appeared inconsistent because of abnormal length of the amino acid sequence compared with homologues in other species, multiple assignments for the same protein for sequences directly next to each other, and annotations that rather than complete protein sequences only represented a signal sequence or the absence of such. Such annotations were therefore compared with their homologues in the *A. actinomycetemcomitans* serotype c strain D11S-1 genome [90]. When the present manuscript was under revision, the D7S whole genome shotgun project was superseded by the complete genome record (CP003496).

All annotated ORFs were analyzed for the presence of protein transport systems using the Transporter Classification Database [110] and combined with the results from TransportDB (http://www.membranetransport.org/) [111], the ABCdb database for ABC transporters in Archea and Bacteria (http://www-abcdb.biotoul.fr/) and Prosite searches with (PS50893, PS51012, PS50928, PS50990, PS50929, PS00211) for ABC transporter motifs.

The presence of signal sequences was determined using SignalP (http://www.cbs.dtu.dk/services/SignalP/) [112], Phobius (http://phobius.sbc.su.se/) [113], Predisi (http://www.predisi.de/) [114] and PSortb (http://www.psort.org/psortb/) [115] and results were interpreted using a most-votes approach. LipoP (http://www.cbs.dtu.dk/services/LipoP/) [116] was used to identify proteins with a lipobox. TatP (http://www.cbs.dtu.dk/services/TatP/) [112] and TatFind (http://signalfind.org/tatfind.html) [117] were used to identify proteins secreted via the Tat pathway. In addition the PattinProt software (http://npsa-pbil.ibcp.fr/cgi-bin/npsa_automat.pl?page  = /NPSA/npsa_pattinprot.html) was used to search for Tat and other motifs. The probable secondary structure of the proteins was analyzed by the search for beta-barrels with BOMP (http://services.cbu.uib.no/tools/bomp) [118] and with TMB-Hunt (http://bmbpcu36.leeds.ac.uk/~andy/betaBarrel/AACompPred/aaTMB_Hunt.cgi) [119]. The presence of transmembrane alfa-helixes was inspected with the TMHMM (http://www.cbs.dtu.dk/services/TMHMM/) [120], ThumbUP (http://sparks.informatics.iupui.edu/Softwares-Services_files/thumbup.htm) [121] and Phobius. Tools to predict subcellular localization were SosuiGramN (http://bp.nuap.nagoya-u.ac.jp/sosui/) [122], Cello 2.5 (http://cello.life.nctu.edu.tw/) [123], PsortB, and a most-votes analysis. If SosuiGramN, Cello 2.5, and PsortB all predicted different subcellular localizations for a particular protein, the protein was grouped into “three different predicted subcellular localizations”.

## Supporting Information

Figure S1 Silver-stained SDS-PAGE of the secretome preparations (protein concentration ∼0.75 µg/µl) of strain D7S grown as biofilm. Samples (∼20 µg protein) of secretome preparation 1 (A), and preparation 2 (B), were applied on the gel. The indicated gel bands were excised from the gel and processed for LC-MS/MS analysis. The approximate locations of the protein bands (10 to 150 kDa) of the prestained molecular weight marker are indicated.
(TIF)

Figure S2 Distribution of the identified *A. actinomycetemcomitans* strain D7S secretome proteins according to their predicted subcellular localization. One hundred and seventy-nine different proteins were identified by LC-MS/MS.
(TIF)

Figure S3 Immunoblot detection of GroEL and PAL released by *A. actinomycetemcomitans* strain D7S grown as biofilm. Lane 1: a representative, filtered and concentrated supernatant sample (protein concentration ∼0.75 µg/µl; ∼5 µg protein applied on the gel) was applied in lane 1. The following whole cell preparation samples (protein concentration ∼1 µg/µl; 10 µg loaded each) were used as controls: lane 2. D7S, lane 3. D7S-p (PAL-deficient derivative of D7S). Polyclonal antisera specific for *E. coli* GroEL, and *A. actinomycetemcomitans* PAL were used for immunoblot detection. Sizes (kDa) of proteins in the prestained molecular weight marker (M) are indicated.
(TIF)

Table S1 Proteins identified by LC-MS/MS in two independent secretome preparations of *A. actinomycetemcomitans* strain D7S grown as biofilm.
(XLS)

Table S2 Identification of virulence-related proteins among the proteins predicted to be secreted by *A. actinomycetemcomitans* strain D7S using *in silico* analysis. (A) Proteins containing a lipobox. (B) Proteins predicted to form a beta-barrel. (C) Proteins for which the subcellular localization was predicted based on a most votes analysis.
(XLS)

Table S3 Protein secretion machinery components encoded in the genome of *A. actinomycetemcomitans* strain D7S. (A) Sec and Tat secretion machinery components. (B) Secretory ABC transporter system components. (C) Secretion systems tentatively involved in the transport of proteins across the outer membrane.
(XLS)
