# Supplementary figures and images for: Proteomics of Protein Secretion by Aggregatibacter actinomycetemcomitans
Source: PLoS One. 2012 Jul 25;7(7):e41662. doi: 10.1371/journal.pone.0041662 (PMC3405016; doi:10.1371/journal.pone.0041662)

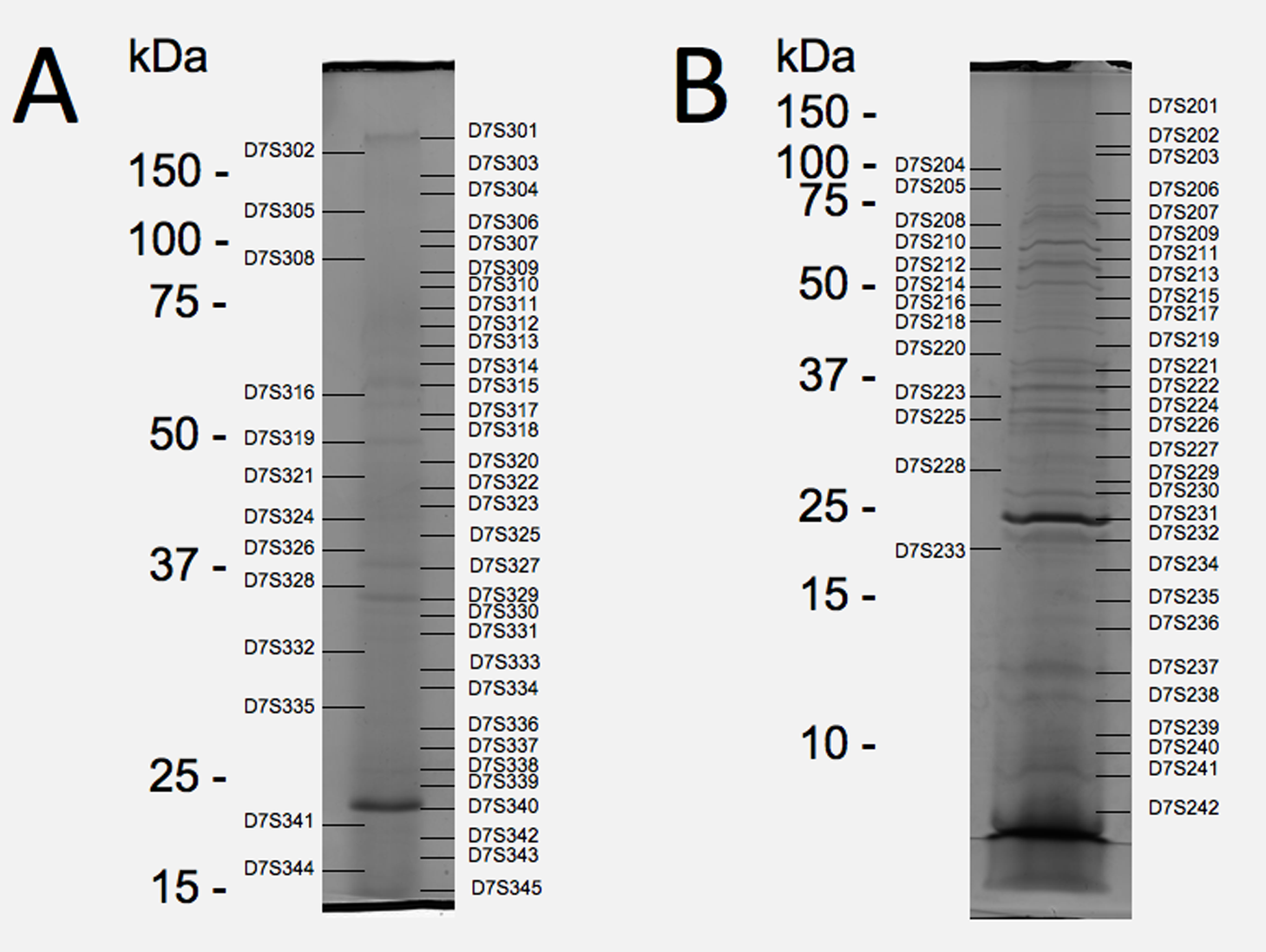

Supplement: Figure S1 — Silver-stained SDS-PAGE of the secretome preparations (protein concentration ∼0.75 µg/µl) of strain D7S grown as biofilm. Samples (∼20 µg protein) of secretome preparation 1 (A), and preparation 2 (B), were applied on the gel. The indicated gel bands were excised from the gel and processed for LC-MS/MS analysis. The approximate locations of the protein bands (10 to 150 kDa) of the prestained molecular weight marker are indicated. (TIF) [file pone.0041662.s001.tif]

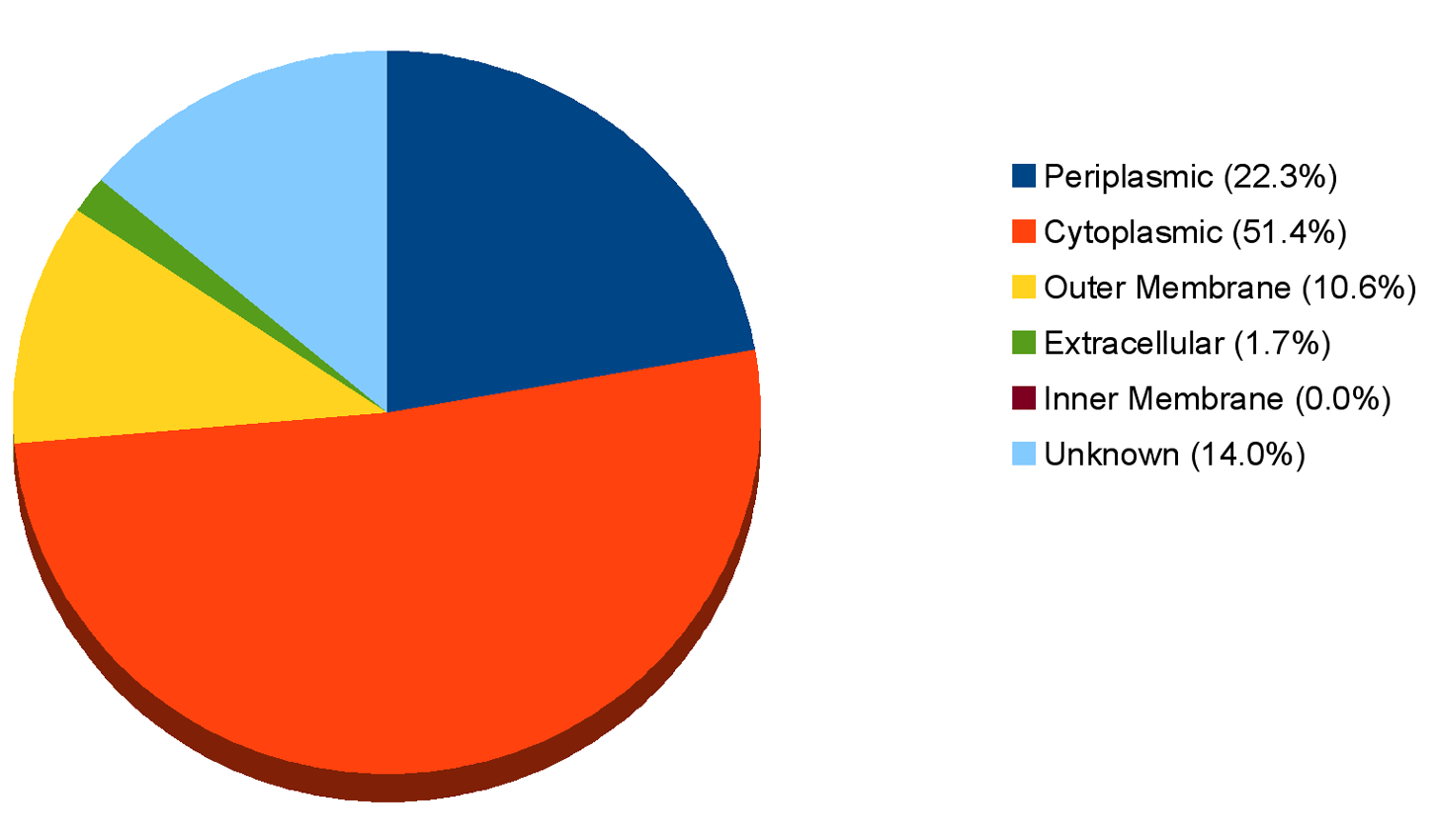

Supplement: Figure S2 — Distribution of the identified A. actinomycetemcomitans strain D7S secretome proteins according to their predicted subcellular localization. One hundred and seventy-nine different proteins were identified by LC-MS/MS. (TIF) [file pone.0041662.s002.tif]

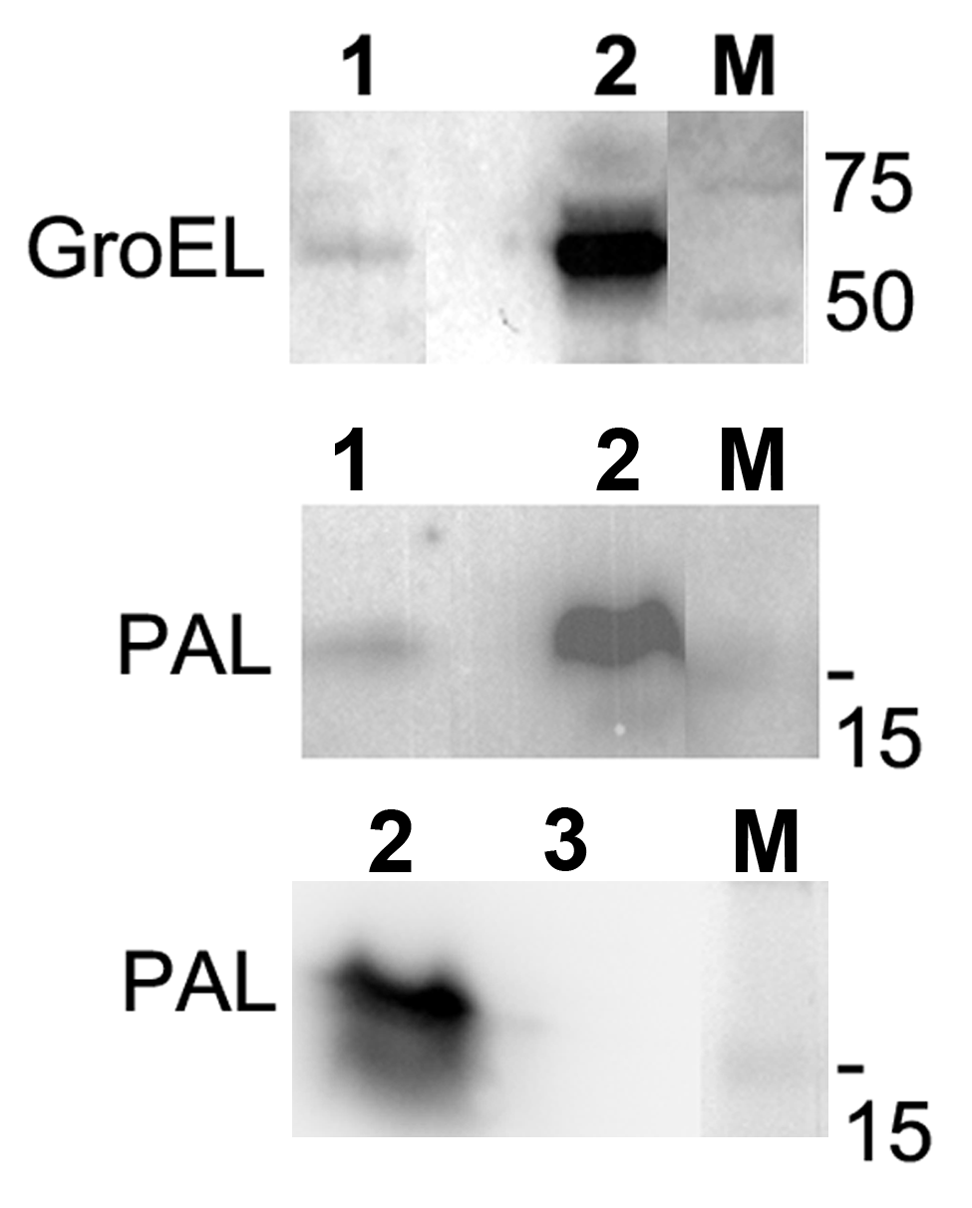

Supplement: Figure S3 — Immunoblot detection of GroEL and PAL released by A. actinomycetemcomitans strain D7S grown as biofilm. Lane 1: a representative, filtered and concentrated supernatant sample (protein concentration ∼0.75 µg/µl; ∼5 µg protein applied on the gel) was applied in lane 1. The following whole cell preparation samples (protein concentration ∼1 µg/µl; 10 µg loaded each) were used as controls: lane 2. D7S, lane 3. D7S-p (PAL-deficient derivative of D7S). Polyclonal antisera specific for E. coli GroEL, and A. actinomycetemcomitans PAL were used for immunoblot detection. Sizes (kDa) of proteins in the prestained molecular weight marker (M) are indicated. (TIF) [file pone.0041662.s003.tif]
